# Short‐term decline of *Castanopsis fargesii* adult trees promotes conspecific seedling regeneration: The complete process from seed production to seedling establishment

**DOI:** 10.1002/ece3.6719

**Published:** 2020-09-09

**Authors:** Li Huang, Lihua Zhou, Jingmei Wang, Cheng Jin, Siwei Hu, Shenhua Qian, Dunmei Lin, Liang Zhao, Yongchuan Yang

**Affiliations:** ^1^ Key Laboratory of the Three Gorges Reservoir Region’s Eco‐Environment Ministry of Education Chongqing University Chongqing China; ^2^ Joint International Research Laboratory of Green Building and Built Environment Ministry of Education Chongqing University Chongqing China

**Keywords:** *Castanopsis fargesii*, forest decline, seed fate, seed production, seedling recruitment, seedling regeneration

## Abstract

Declining forests usually face uncertain regeneration dynamics and recovery trajectories, which are challenging to forest management. In this study, we investigated the decline pattern of *Castanopsis fargesii* and examined the effects on conspecific seedling regeneration. We found that 61.45% of adult individuals were in decline and the smaller DBH size classes of trees (10–40 cm) had a greater probability of decline. Most of the intermediate decline (94.52%) and nondecline individuals (95.23%) did not worsen, and the crowns of 21.91% of the intermediate decline trees were recovered during 2013–2018. Adult tree decline had a negative effect on seed production (mean mature seed density of nondecline, intermediate decline, and high decline individuals was 167.3, 63.3, and 2.1 seeds/m^2^, respectively), but no effect on key seed traits. The seed survival rate of declining trees was greater than that of nondeclining trees at both the seed production and seed dispersal stages. The seed to seedling transition rates in canopy gaps, decline habitats, and nondecline habitats were 7.94%, 9.47%, and 109.24%, respectively. The survival rate and height growth of newly germinated seedlings were positively correlated with the light condition, which was notably accelerated in the canopy gaps. Taken together, these results indicate that the reduction in seed production of some adult trees had a weakly negative effect on new seedling recruitment, while the improved environmental condition after the decline significantly enhanced the survival and growth of both advanced and new germinated seedlings. Looking at the overall life history, the short‐term defoliation and mortality of some *C. fargesii* adult trees can be regarded as a natural forest disturbance that favors conspecific seedling regeneration. High‐intensity management measures would be unnecessary in cases of an emerging intermediate decline in this forest.

## Graphical abstract

The reduction in seed production of some adult trees caused by decline had a weakly negative effect on new seedling recruitment, while the improved environmental condition after the decline significantly enhanced the survival and growth of both new and advanced seedlings. Looking at the overall life history, the short‐term defoliation and mortality of some *Castanopsis fargesii* adult trees can be regarded as a natural forest disturbance that favors conspecific seedling regeneration.

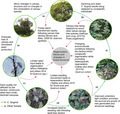

## 1 INTRODUCTION

Widespread tree defoliation and mortality rates have increased considerably in recent decades (Allen et al., 2010; Cohen et al., 2016; Jung, Blaschke, & Oßwald, 2000; van Mantgem et al., 2009; Vilà‐Cabrera, Martínez‐Vilalta, Galiano, & Retana, 2013; Vindstad, Jepsen, Ek, Pepi, & Ims, 2019). Although the underlying causes for this phenomenon are not known for certain, they are frequently associated with global environmental changes, such as more intense and frequent droughts, higher temperatures, outbreaks of invasive pests and pathogens, or the interactions among these factors (Carnicer et al., 2011; Heitzman, Grell, Spetich, & Starkey, 2007; Kayes & Tinker, 2012; Klutsch et al., 2009; Kurz et al., 2008; Loo, 2009; Thomas, Blank, & Hartmann, 2002; Vindstad et al., 2019). These factors share a common feature in that they tend to induce species selection and individual removal, leading to changes in the species composition of the community in the short term in conifer‐ or hardwood‐dominated forests (Aynekulu et al., 2011; Collins, Rhoades, Hubbard, & Battaglia, 2011). The management of declining forests has attracted the attention of scientists, forestry practitioners, and policymakers; however, our current understanding of management strategies for declining forests is still limited.

Most of the recent studies have addressed regeneration dynamics in declining forests with a focus on the survival and growth of advanced seedlings (Ibáñez, Gómez‐Aparicio, Ávila, Pérez‐Ramos, & Marañón, 2017; Mertelmeyer, Jacobi, Mueller‐Dombois, Brinck, & Boethmer, 2019) to suggest that declining forests tend to face an uncertain future and recovery trajectory (Galiano, Martínez‐Vilalta, & Lloret, 2010; Kayes & Tinker, 2012; Palik, Ostry, Venette, & Abdela, 2012; Redmond & Barger, 2013; Vindstad et al., 2019). Tree defoliation and mortality can induce a series of changes in local environmental conditions (e.g., higher solar radiation at the forest floor, more extreme temperatures, and accelerated nutrient fluxes in the soil) that alter the probability of establishment of conspecific tree seedlings (Ibáñez et al., 2017; Kayes & Tinker, 2012; Redmond & Barger, 2013). For example, an increase in radiation levels and drought stress in the gaps that open after tree death sometimes preclude the establishment of late‐successional shade‐tolerant species, and indirectly favor pioneer, drought‐tolerant species that can lead to changes in the forest structure (Diskin, Rocca, Nelson, Aoki, & Romme, 2011; Ibáñez et al., 2015; Redmond & Barger, 2013). Moreover, the trajectories of recovery after drought‐ or insect‐driven tree mortality may depend on advanced regeneration being established prior to the disturbance (Collins et al., 2011; Kayes & Tinker, 2012; Redmond & Barger, 2013). For example, tree dieback in mature forests can release suppressed saplings of shade‐tolerant species, which allows the late‐successional species to continue dominating the stands and indirectly limiting the establishment of light‐demanding pioneer species that would otherwise be typical of the disturbed sites (DeRose & Long, 2010). These examples illustrate the complexities inherent to the postmortality regeneration dynamics, where species with different niches and seedlings at different age stages tend to respond differently (Galiano, Martínez‐Vilalta, Eugenio, Granzow‐de la Cerda, & Lloret, 2013).

New seedling recruitment dynamics is a little studied aspect of declining forests, despite the knowledge being important for forest management (McDowell, Ryan, Zeppel, & Tissue, 2013). New seedling recruitment is a multiphase process involving seed production, seed dispersal, seed germination, seedling emergence, and early survival of seedlings (Wang & Smith, 2002; Yang, Huang, Qian, & Fukuda, 2015). The accurate quantification of the impact of canopy tree decline at each stage of the seedling recruitment process is significant for predicting the long‐term dynamics and management of declining forests. Although earlier studies have shown that adult tree defoliation can cause a reduced seed production due to a smaller gain in carbohydrate (Nakajima & Ishida, 2014; Palacio, Hoch, Sala, Körner, & Millard, 2014; Vilà‐Cabrera, Martínez‐Vilalta, & Retana, 2014), little is known about the possible effects on seed traits (e.g., seed mass and nutrient content) that play decisive roles in seed survival and germination and seedling early competition (Lebrija‐Trejos, Reich, Hernández, & Wright, 2016). At the seed dispersal stage, predation and fungal infections are the main seed‐killing agents that determine the success of the seed to seedling transition (Tomita, Hirabuki, & Seiwa, 2002; Yang et al., 2015). Both of these factors are negatively density‐dependent and easily influenced by local environmental conditions (e.g., soil moisture and canopy openness) postdecline (Yang et al., 2015). Nevertheless, we have scant knowledge of the effects of tree defoliation and the subsequent changing environmental conditions on the fate of seeds at the seed dispersal stage. Accordingly, studies need to be conducted to reveal the underlying ecological mechanisms involved in the process of new seedling input postforest decline.

Subtropical evergreen broad‐leaved forests are recognized as an important global vegetation formation type that contributes to the sustainable development of subtropical regions (Song & Da, 2016). In recent years, cases of *Castanopsis fargesii* (one of the most widely distributed dominant species in evergreen broad‐leaved forests) decline have been seen along the Yangtze River. The decline of *C. fargesii* dominated forests would pose a great threat to the integrity of the forests and would further influence the supply of ecosystem services. Although *C. fargesii* is recognized as a late‐successional and shade‐tolerant species (Cornelissen, 1993; Yang et al., 2015), its regeneration depends on the understory light condition. Since 2008, a serious decline of *C. fargesii* has been found in the Jinyun Mountain National Nature Reserve in the middle and upper reaches of the Yangtze River. Sano et al. (2014) and Takahashi et al. (2014) examined pathogenic bacterial infections as possible causes of *C. fargesii* adult tree decline. In the current study, we address the pattern of decline of *C. fargesii* adult trees, and assess the effects of the adult tree decline on seedling establishment, including seed production, seed fate at the seed dispersal stage, seedling emergency, and seedling survival and growth. We tested the following three predictions: (a) The decline of *C. fargesii* has a negative effect on seed production but has no effect on seed traits. (b) The seeds of declining adult trees have a higher survival rate at the seed production stage and seed dispersal stage, which would increase the seed to seedling transition rate. (c) Improved light conditions for establishment beneath the canopies of declining trees contribute to both newly germinated seedlings and advanced seedling survival and growth. By testing these predictions, we can confirm whether or not the defoliation and mortality of *C. fargesii* adult trees can be identified as short‐term natural disturbances that would benefit short‐term conspecific seedling regeneration.

## 2 MATERIALS AND METHODS

### 2.1 Study site and study species

We conducted this study on Jinyun Mountain (29°49′N, 106°20′E), a national nature reserve in Chongqing in Southwestern China. The site has a subtropical monsoon climate with an annual mean temperature of 13.6°C, a mean January temperature of 3.1°C, and a mean August temperature of 24.3°C. The annual mean precipitation and relative humidity are 1,611.8 mm and 87%, respectively (Yang et al., 2014). Vegetation on Jinyun Mountain is composed of typical middle‐subtropical evergreen forests, which are codominated by *C. fargesii*, *Machilus pingii*, *Symplocos setchuensis*, and *Castanopsis carlesii* var. *spinulosa* (Yang et al., 2014, 2015).

Adult *C. fargesii* trees can attain 30 m in height. Mean seed mass of *C. fargesii* is about 0.55 g and ranged from 0.1 to 1.8 g (Yang et al., 2015). Seeds are primary dispersed by gravity and secondary dispersed by rodents such as squirrels and mice (Du, Guo, Gao, & Ma, 2007). The fruits ripen from October to November, and the peak seed rain occurs between October and November (Yang et al., 2015). The emergence of seedlings usually starts in July and August and ends in late November.

### 2.2 Fieldwork

**Figure 1:**
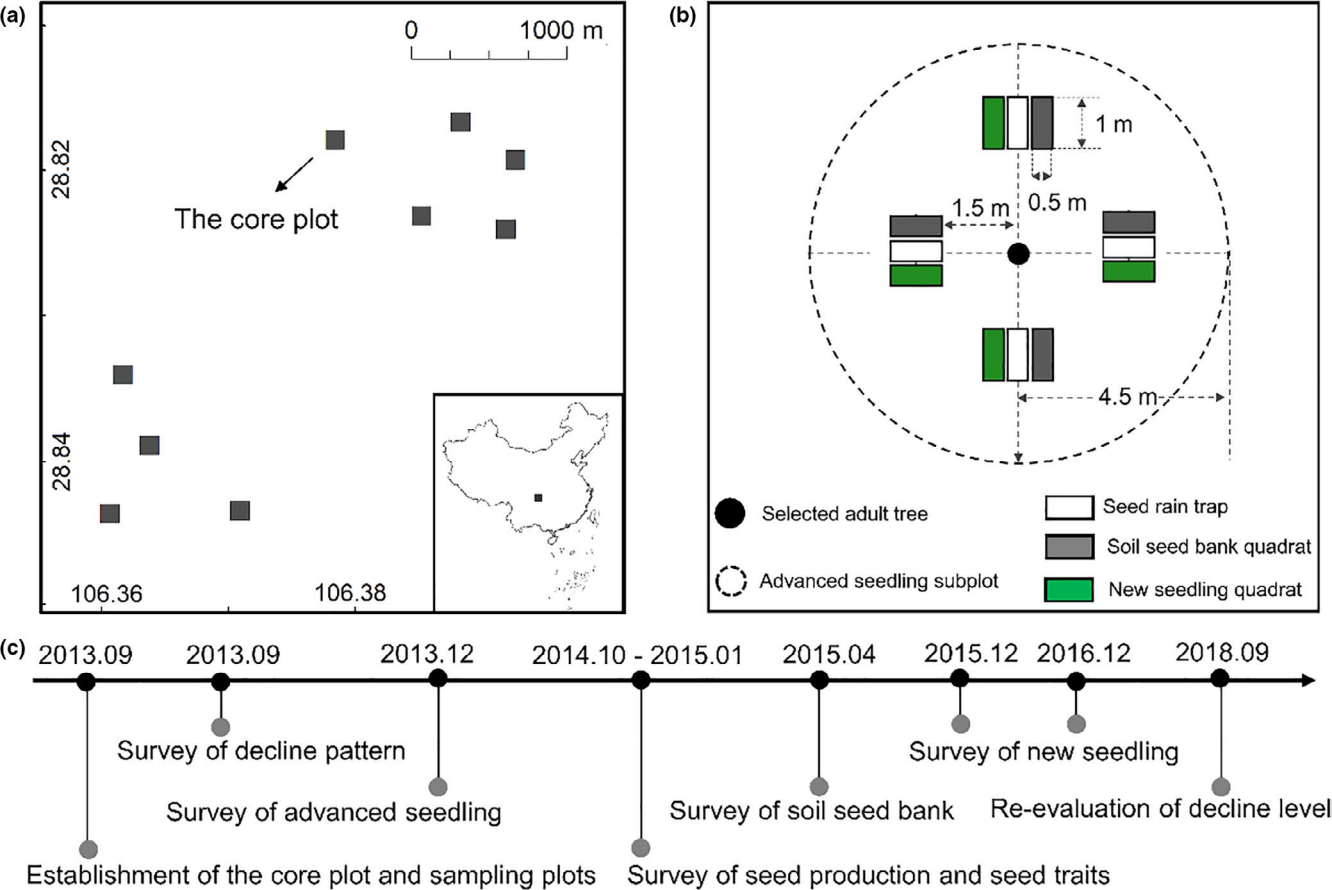
(a) Geographic distribution of study plots; (b) schematic diagram of advanced seedling subplots and quadrats and (c) the timeline diagram of surveys

In September 2013, we established a 0.5 ha (50 m × 100 m) core monitoring plot and eight 30 m × 30 m sampling plots that were dominated by *C. fargesii* to monitor the long‐term dynamics of declining forests (Figure 1a). Detailed information about the core plot and eight sample plots is summarized in Table 1. This sampling design was adopted because evergreen broad‐leaved forests generally occur in scattered patches, making it difficult to find a large area of continuously distributed *C. fargesii* forests at our study site. Monitoring was done to assess: (a) the decline patterns and trends of *C. fargesii* over time; (b) changes in seed production and seed traits; (c) the recruitment dynamics of new seedlings; and (d) the impacts of adult tree decline on the advanced seedling distribution pattern and growth.

**TABLE 1:** Characteristics of the core and sample plots

Plots	Plot size (m × m)	Altitude (m)	Inclination (°)	Number of species	Number of trees	BA/m^2^	Total BA (m^2^)	RBA of *C. fargesii* (%)
C1	50 × 100	825	20	74	1,318	0.0065	32.72	48.31
F1	30 × 30	890	20	29	183	0.0025	2.24	67.86
F2	30 × 30	900	15	25	203	0.0043	3.9	57.44
F3	30 × 30	880	25	24	383	0.0032	2.9	35.86
F4	30 × 30	870	30	21	183	0.0034	3.09	27.83
Q1	30 × 30	840	10	18	71	0.0024	2.19	77.17
Q2	30 × 30	830	15	19	122	0.0041	3.68	81.52
Q3	30 × 30	800	15	20	212	0.0034	3.03	39.27
Q4	30 × 30	790	20	31	307	0.0056	5.03	58.25

#### 2.2.1 Survey of the decline pattern of Castanopsis fargesii

In September 2013, we examined the decline patterns of *C. fargesii* adult trees within the core monitoring plot and the eight sampling plots. Within each plot, we tagged and measured diameter‐at‐breast height (DBH) and tree height (H) of all woody trees ≥1.3 m tall. For *C. fargesii* adult trees (H ≥ 8 m; DBH ≥ 10 cm) and saplings (1.3–8 m) in all plots, we determined the vigor levels based on the percentage of crown defoliation, using a semi‐quantitative scale (Linares & Camarero, 2012). Six tree vigor levels were defined for this study: L0: dead tree; L1: 81%–100% crown defoliation; L2: 61%–80% crown defoliation; L3: 41%–60% crown defoliation; L4: 21%–40% crown defoliation; and L5: 0%–20% crown defoliation. We classified L0–L1 as a high level of decline, L2–L3 as an intermediate level of decline, and L4–L5 as nondecline. In September 2018, we again determined the vigor levels of all of the adult trees that we had marked in 2013 to evaluate their decline trends.

#### 2.2.2 Survey of advanced seedlings

In September 2013, we set up 30 circular advanced seedling (existing before our study) subplots (radius = 4.5 m, determined by the average radius of healthy *C. fargesii* canopies, area = 63.585 m^2^, total area = 1,907.55 m^2^) with the surrounding 30 adult trees in the core monitoring plot (Figure 1b). We selected individuals without any other adult individuals within 10 m to ensure that the crowns of any two individuals were not overlapping. The habitats of the 30 subplots were categorized as NDE, DE, and CGA based on the decline level of the selected adult trees: (a) NDE, under nondeclining *C. fargesii* canopies (*n* = 11); (b) DE, under intermediate declining *C. fargesii* canopies (*n* = 10); (c) CGA, in canopy gaps that were established following the death or severe defoliation of *C. fargesii* adult trees (*n* = 9).

In December 2013, all advanced seedlings (H < 1.3 m, existing before this study) in these subplots were investigated by determining their ages (by counting the bud scars on the seedlings) and measuring the heights. We defined three seedling stages based on the ages of the advanced seedlings in each circular subplot: small seedling, age ≤5 years; intermediate seedling, 5–10 years; large seedling, age >10 years (Yang et al., 2015). The annual ring analysis showed that the decline of *C. fargesii* adult trees and the formation of canopy gaps in the study plots were earlier than 2000. Consequently, most seedlings in the CGA habitats were established after the decline.

#### 2.2.3 Survey of seed production and seed traits

We investigated seed production of the 30 selected *C. fargesii* adult trees using seed traps. The seed traps were made of a funnel of polyethylene cloth (1‐mm mesh), with a receiving area of 0.5 m^2^ (1 m × 0.5 m) and a height of 1 m above the ground (Yang et al., 2015). Considering the terrain, crown shape, and vine coverage, we set up two or three seed traps in each subplot at a distance of 1.5 m from each adult tree (72 seed traps in total, Figure 1). We fully considered the distance between two selected adult trees to ensure that a certain distance (>3 m) was present between the seed traps and the adjacent adult tree canopies. In addition, *C. fargesii* seeds are about 0.5 g, and the horizontal dispersal distance is very close by seed rain. Therefore, the risk that seeds in the seed traps had come from adjacent adult trees was very low.

**Figure 2:**
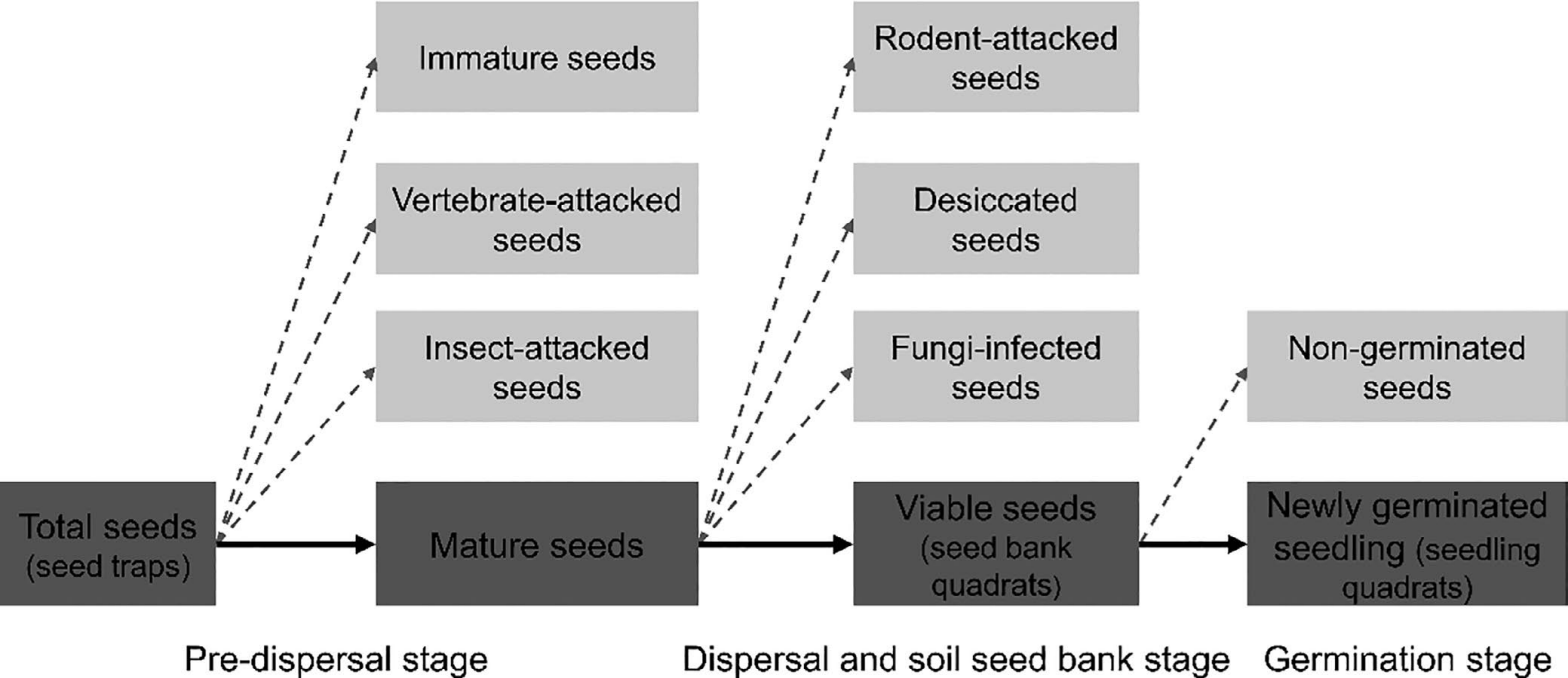
Seed fate pathways of *C. fargesii* in seed to seedling transition process

The seeds that accumulated in the traps were collected at 1‐week intervals from October 2014 to January 2015 (until no seeds accumulated for more than 2 weeks). The collected seeds were air‐dried and sorted. We categorized the collected seeds as mature seeds (intact, without any injury), immature seeds (the embryo and cotyledons were incompletely developed), vertebrate‐attacked seeds (mainly eaten by squirrels before falling), and insect‐attacked seeds (suffered predation by invertebrate moth larvae with obvious wormholes on the seed surface) (Figure 2), and counted the total number of seeds in each category.

We combined the mature seeds of each adult tree collected every week. Among the combined seeds, we randomly selected 30 intact mature seeds from each of the 11 nondeclining adult trees and 10 intermediate declining adult trees to measure their seed traits, including fresh weight (seed mass), nutritional (starch), and defensive (cellulose and tannin) material properties. We did not measure the seed traits of high declining adult trees, because the collected seeds were too few to sample. Starch content was estimated using the optical rotation method following the national standard (NY/T11‐1985) for cereals. Cellulose content was estimated using the acid and alkali washing method following the national standard (NY/T13‐1986) for cereals (Wang, Zhang, Zhang, Li, & Yi, 2016). The tannic acid content was determined using the spectrophotometry method following the national standard (GB/T15686‐2008) for sorghum (Wang & Chen, 2012).

#### 2.2.4 Survey of new seedling recruitment and environmental conditions

In April 2015 (before seed germination), we set up a 0.5 m^2^ (1 m × 0.5 m) seed bank quadrat at the right side, close to the seed trap to investigate the seed fate at the seed dispersal stage (a total of 72 seed bank quadrats; Figure 1b). We collected the humus and topsoil of each soil seed bank quadrat and then separated and sorted *C. fargesii* seeds from the soil samples. The collected seeds were categorized into four groups after a careful examination: (a) viable seeds (already had an elongated radicle or healthy embryos and cotyledons); (b) fungi‐infected seeds (killed by fungal infection, where the embryo and/or cotyledons were covered with hyphae and decayed); (c) desiccated seeds (killed by desiccation, where the roots and/or cotyledons were withered and brown), and (d) rodent‐attacked seeds (mainly killed by rodents after falling) (Figure 2). This classification was according to Tomita et al. (2002) and Yang et al. (2015).

In December 2015 (after seedling emergence), we established a 0.5 m^2^ (1 m × 0.5 m) new seedling quadrat at the left side, close to each of the 72 seed traps (Figure 1b). We censused the newly germinated seedlings (hereafter, “new seedlings”) within each seedling quadrat and measured their stem heights. In December, 2016, we revisited the seedling quadrats to investigate seedling survival and stem height.

Within each seedling quadrat, we measured the light conditions and soil conditions in 2016. We measured the photosynthetic photon flux density (PPFD) at 1.3 m above the forest floor using a LightScout 6 sensor quantum bar (LightScout; Spectrum Technologies, Inc.) and the in situ soil temperature and moisture using an HH2 moisture meter (Delta‐T devices) at the surface soil layer (0–11 cm depth) in four seasons. Soil samples from each seedling quadrat were collected from 6 to 7 August 2016. In each seedling quadrat, the litter layer was removed and three cores of topsoil were taken with a 5‐cm‐diameter corer at a depth of 0–10 cm. These were mixed and stored in plastic bags for transport to the laboratory on the same day. Soil clods were broken by hand into smaller pieces, air‐dried with a fan under shade for 30 days, and then ground and sieved. Soil pH was measured in water (soil: deionized water ratio = 1:2.5) with a pH meter (PHS‐3C, Shanghai Precision Scientific Instrument Co., Ltd.). Total carbon (C) and nitrogen (N) concentrations were measured by dry combustion using an elemental analyzer (MACRO Cube Elemental Analyzer). Phosphorus (P) was measured using inductively coupled plasma emission spectroscopy (ICP‐OES; Therma Jarrel‐Ash, IRIS Advantage) after digestion with concentrated HNO_3_ and 30% H_2_O_2_.

### 2.3 Data analysis

The seed densities (seeds/m^2^) of each seed trap were calculated as *x_i_*/*y_i_* × 100%, where *x_i_* was the total seeds collected from a seed trap, and *y_i_* was the area of a seed trap. The seed density of an adult tree was calculated as the mean value of all seed traps set under the tree.

We used a one‐way ANOVA to test differences in seed production (seed density) and seed traits for the declining and nondeclining trees. We used Tukey's HSD to test differences in stem height and density of the advanced and newly germinated seedlings at each stage across different habitats. Before the analysis, all data were tested for normal distribution using the Shapiro–Wilk test and homogeneity was tested using the Breusch–Pagan test. Data for seed production were log‐transformed to accomplish normality and homoscedasticity assumptions.

We tested the effects of the environmental conditions of the seedling quadrats on 2‐year survival and stem height of newly germinated seedlings using a generalized linear mixed model (GLMM). For the 2‐year survival of seedlings, we used a binomial family, logit link model with living or dead seedlings (0/1) as the response variable, environmental conditions as the fixed effects, and tree ID as the random effect. For stem height of seedlings, we used a Gaussian distribution, log‐link model with seedling stem height of seedlings as the response variable, environmental conditions as the fixed effects, and tree ID as the random effect. We used an odds ratio regression to determine the effect of each environmental condition on both seedling 2‐year survival and height growth (Liang et al., 2016). An odds ratio (significance >1) indicated that environmental conditions increased the seedling survival and growth, and an odds ratio (significance <1) indicated that environmental conditions caused seedling mortality and decreased seedling growth.

All statistical analyses were performed in R version 3.5.1 (R Core Team, 2018), with the GLMM regression conducted using the “lme4” package (Kuznetsova, Brockhoff, & Christensen, 2017).

## 3 RESULTS

### 3.1 Patterns and trends of decline

**Figure 3:**
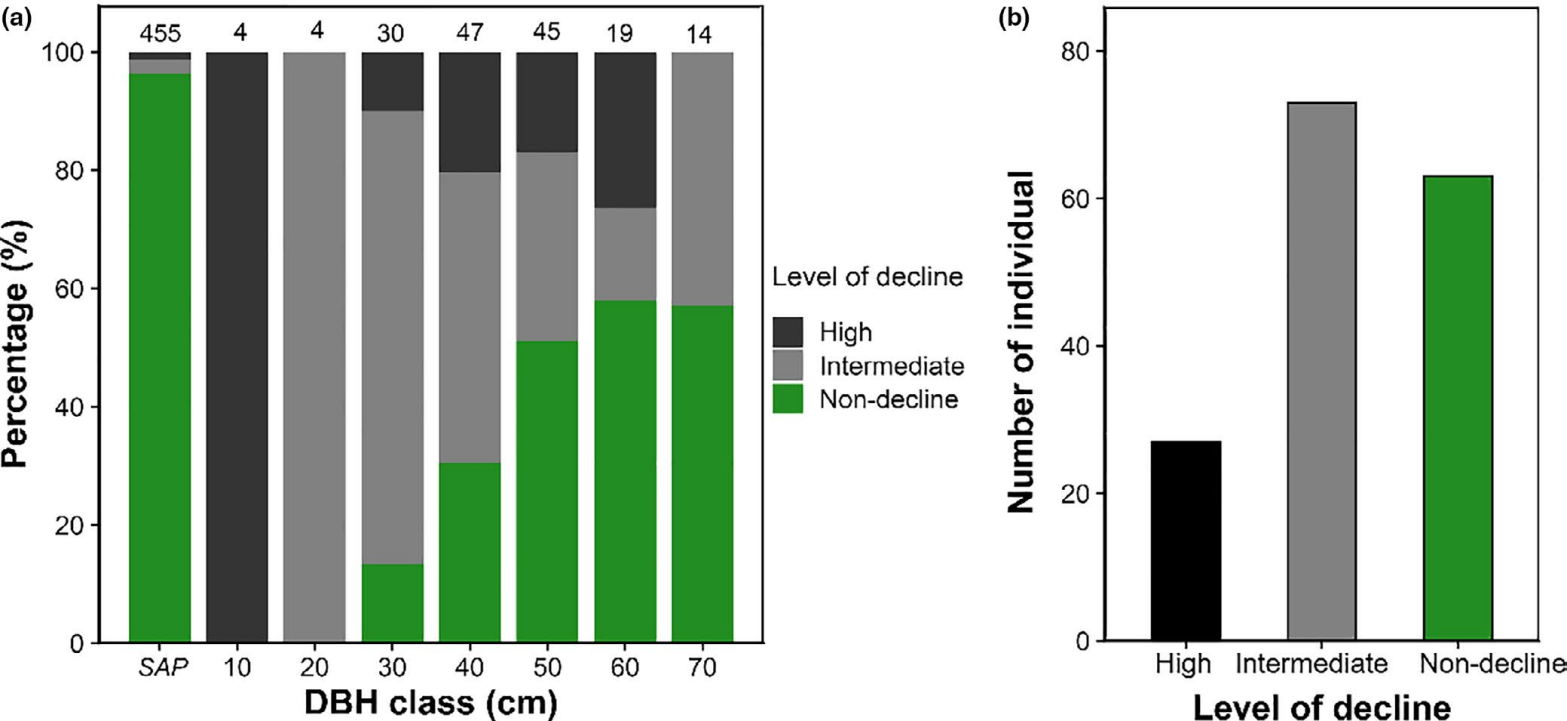
(a) Distribution of percentage decline individuals across DBH size for sapling and adult trees and (b) comparisons of tree abundance in the three decline levels of adult trees. In (a), the numbers above each bar refer to the sample size in each group

In 2013, we recorded 163 adult trees and 454 saplings from the nine plots. Within the 454 saplings, 17 individuals were in decline (3.74% of the total sapling tree count). For the 163 adult trees, 101 individuals were in decline, which included 28 high decline individuals (17.18% of the total adult tree count) and 73 intermediate decline individuals (44.79% of the total adult tree count; Figure 3b). The distribution of percentage decline across size classes suggested that the decline probability varied in size classes. A higher probability of decline occurred in adult trees in the smaller size classes (10–40 cm, around 70%–100%), but the larger size classes also showed a relatively high probability of decline (50–70 cm, around 40%–50%; Figure 3a).

**Figure 4:**
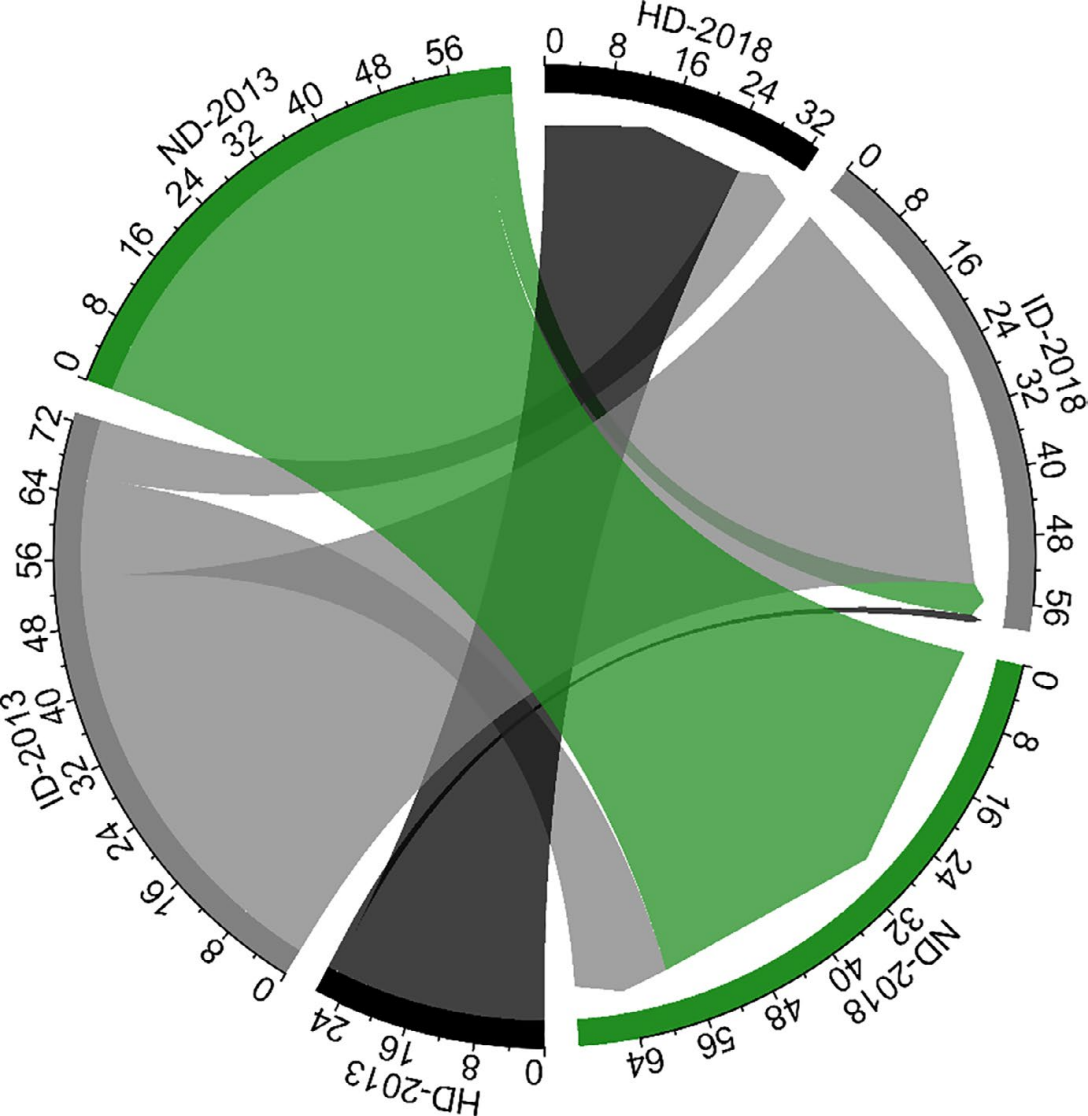
Changes in decline level of the 163 adult trees from 2013 to 2018. ND = nondecline, ID = intermediate level of decline, and HD = high level of decline

In 2018, we re‐evaluated the decline levels of all 163 adult trees. The decline level of 16 individuals decreased, seven trees increased, and 140 trees maintained their decline level. For the seven trees that had an increase in decline level, five were intermediate decline trees in 2013. For the 16 trees that had a decrease in decline level, 15 were intermediate decline trees in 2013. For the 140 trees that maintained their decline level, 26 were high decline trees, 54 were intermediate decline, and 60 were nondecline in 2013 (Figure 4).

### 3.2 Seed production and seed traits

**Figure 5:**
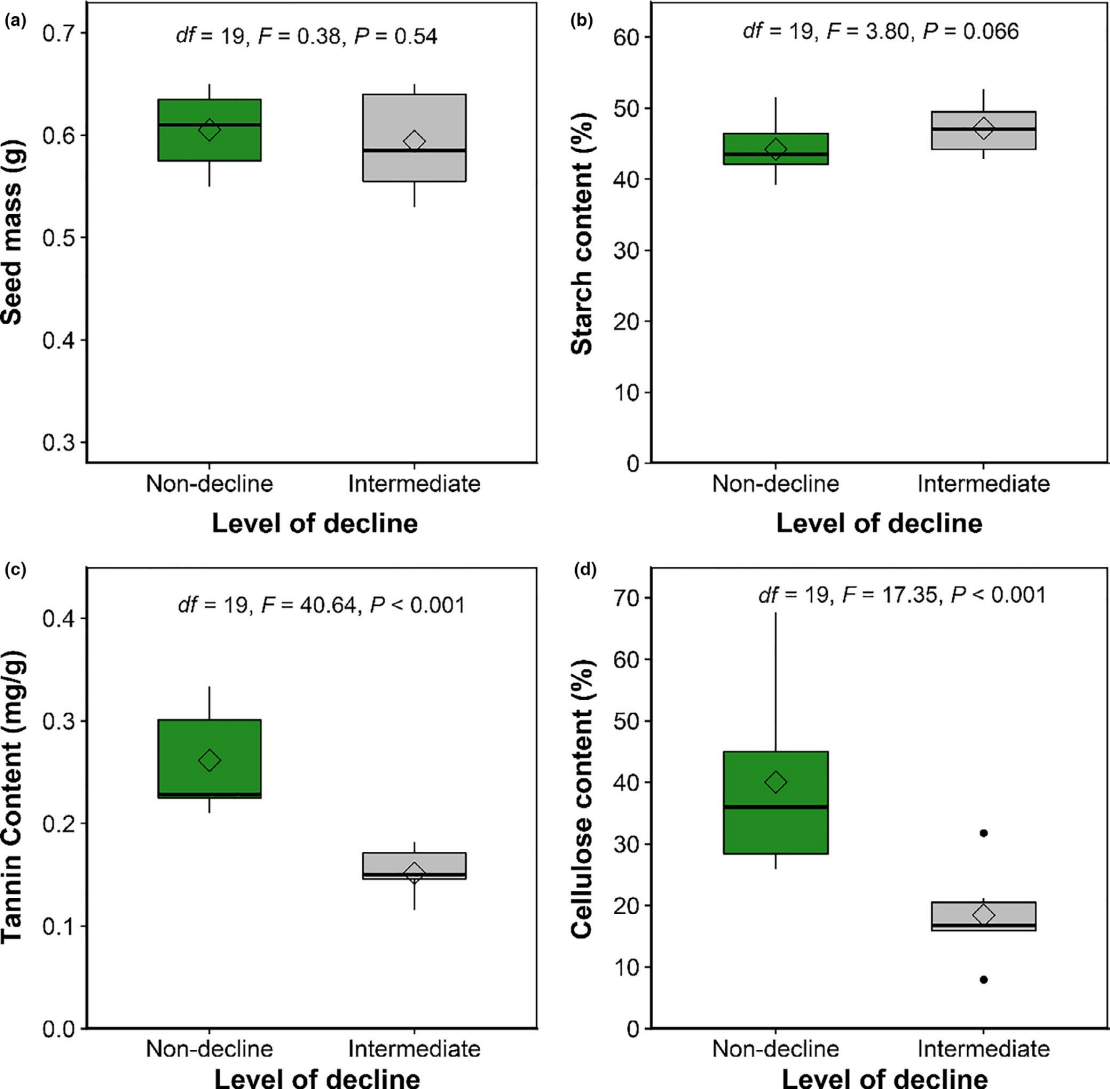
(a) Comparison of seed mass, (b) starch content, (c) tannin content, and (d) cellulose content of seeds produced by intermediate decline and nondeclining individuals

The density of mature seeds, immature seeds, vertebrate‐attacked seeds, and insect‐attacked seeds of declining adult trees was significantly lower than nondeclining trees (Table 2). The proportion of immature seeds, insect‐attacked seeds, and vertebrate‐attacked seeds of declining adult trees was lower than that of nondeclining trees (Table 2). No significant differences were seen in seed mass or starch content of seeds produced by declining or nondeclining adult trees (Figure 5a,b). However, the tannin content and cellulose content of seeds produced by declining adult trees were significantly lower than that produced by nondeclining trees (Figure 5c,d).

**TABLE 2:** Comparison of seed density of the four types of seeds collected from seed traps from the three levels of declining adult trees

Seed type	Seed density (seed/m^2^)		
	Nondecline	Intermediate decline	High decline
Mature seed	167.3 ± 20.5a (32.0)	63.3 ± 8.9b (44.3)	2.1 ± 0.4c
Immature seed	309.4 ± 47.2a (59.3)	73.8 ± 14.2b (51.5)	19.6 ± 3.4c
Vertebrate‐attacked seed	19.1 ± 2.9a (3.7)	2.8 ± 0.4b (2.0)	0.41 ± 0.06c
Insect‐attacked seed	26.3 ± 3.12a (5.0)	3.1 ± 0.5b (2.2)	0.8 ± 0.01c

Note

The different letters indicate significant differences (*p* < .01) based on Tukey's HSD test. The values in brackets represent the percentage of each type of seed.

### 3.3 Seed fate at seed dispersal stage

**Figure 6:**
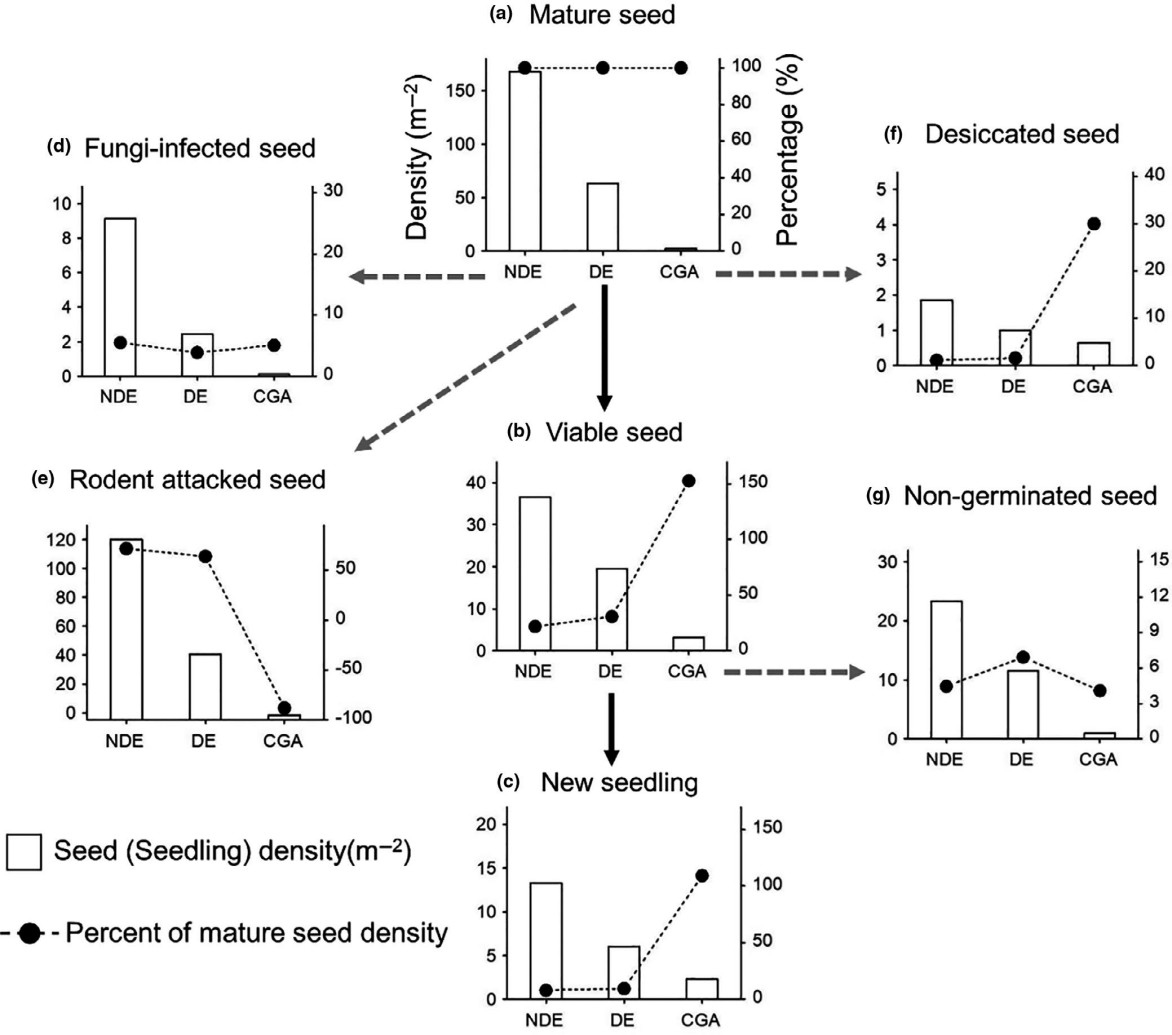
Seed fate of mature seeds in the three habitats. Solid lines show the density of undamaged seeds (mature seeds and viable seeds) or new seedlings (bars in the figure) and their proportion of mature seed density (black solid dots) at each seedling‐established stage. Dashed lines show the density of damaged seeds and their proportion of mature seed density at each seedling‐established stage. NDE = under nondeclining canopies; DE = under declining canopies; and CGA = in canopy gaps

At the seed dispersal stage, the density of rodent‐attacked seeds and fungi‐infected seeds in NDE habitats were significantly higher than DE habitats and CGA habitats (*df* = 27, *F* = 7.24, *p* = .0030, Figure 6e; *df* = 27, *F* = 8.58, *p* = .0013; Figure 6d). The proportion of rodent‐attacked seeds in the NDE habitats was significantly higher than in the CGA and DE habitats (*df* = 27, *F* = 11.2, *p* < .01; Figure 6e). The proportion of desiccated seeds in the CGA habitats was higher than that of the NED and DE habitats (*df* = 27, *F* = 9.71, *p* < .01; Figure 6f). The density of viable seeds in the CGA habitats was 3.24 seed/m^2^, which was 1.5‐times that of mature seeds (Figure 6b). The density of new seedlings in the NDE habitats, DE habitats, and CGA habitats was 13.29 seedlings/m^2^, 6.01 seedlings/m^2^, and 2.29 seedlings/m^2^, respectively. In addition, the seed to seedling transition rate in NDE, DE, and CGA habitats was 7.94%, 9.47%, and 109.24%, respectively. There were more viable seeds than mature seeds, and the transition rate was higher than 100% in CGA habitats because some seeds were transported from other habitats (such as NDE habitats with high seed density) to CGA habitats by seed dispersers and seeds in CGA habitats had a higher survival rate.

### 3.4 Survival and growth of new germinated seedlings

**Figure 7:**
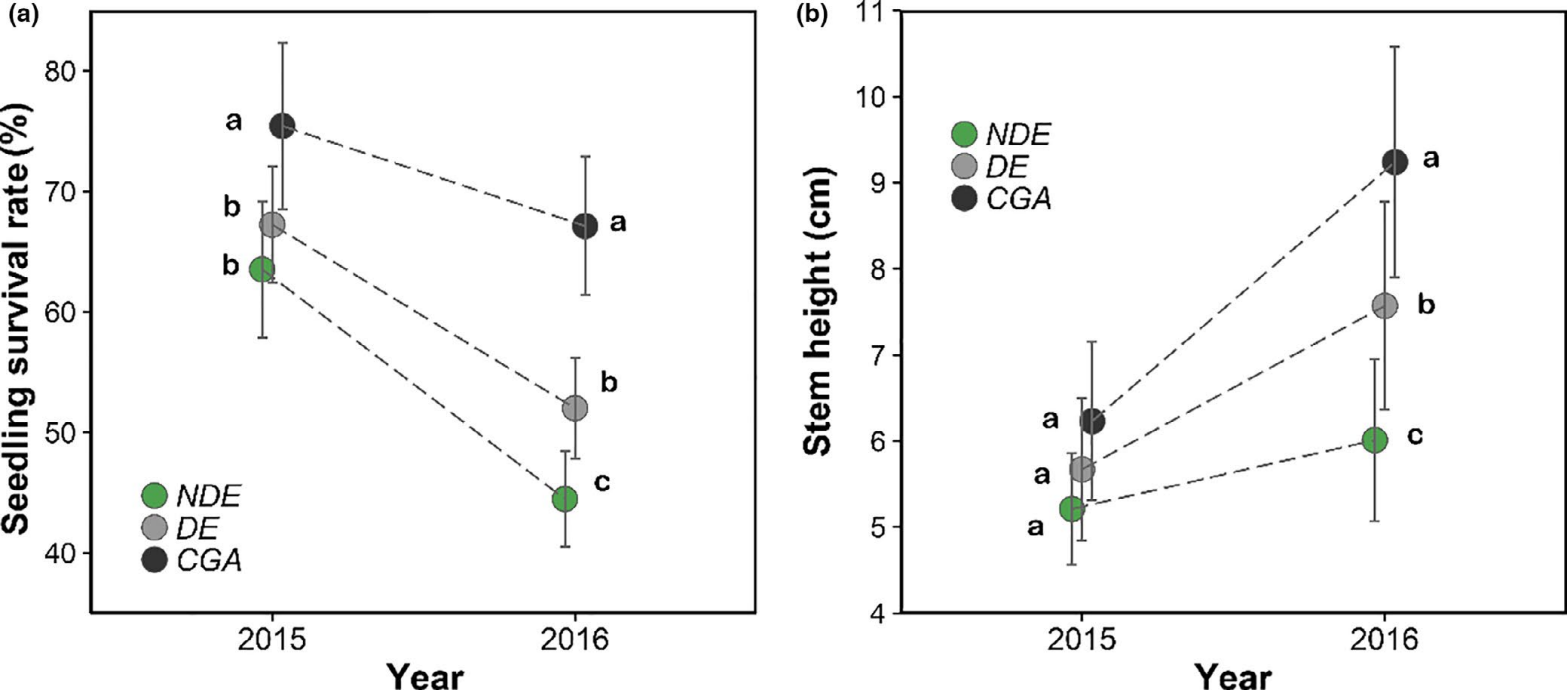
(a) Comparison of survival rates and (b) heights of new seedlings in the three habitats. Error bars are for SE. The different letters above the dots indicate significant differences at *p* < .01, based on Tukey's HSD test. NDE = under nondeclining canopies; DE = under declining canopies; and CGA = in canopy gaps

In 2015, the survival rate of new seedlings in the CGA habitat was significantly higher than that of the NDE and DE habitats. In 2016, the survival rates of new seedlings in the CGA and DE habitats were significantly higher than that of the NDE habitats (Figure 7a). While stem heights did not differ among the three habitats in 2015, stem height of new seedlings in the CGA was higher than that of the DE habitats by 2016, which was higher than that of the NDE habitats (Figure 7b).

PPFD in the DE and CGA habitats was significantly higher than that in the NDE habitats. Total P content of soil in the DE and CGA habitats was significantly lower than that of the NDE habitats. Significant positive correlations were found between PPFD and new seedling survival rates and mean stem heights by 2016 (Table 3), suggesting that the improved light conditions in the CGA habitats promoted the survival and growth of new germinated seedlings.

**TABLE 3:** Comparison of environmental variables for the three types of habitat and the effects of environmental variables on seedling survival and stem height of new germinated seedlings in 2016, based on the GLMM analysis

Environmental variable	Habitat	Odds ratio value			
	NDE	DE	CGA	Survival	Stem height
Soil moisture (v, %)	12.46a	15.76a	15.24a	0.67	1.07
Soil temperature (°C)	25.04a	25.09a	25.09a	0.70	1.23
Total N in the soil (g/kg)	2.01a	1.95a	1.92a	1.35	0.62
Total P in the soil (g/kg)	1.12a	1.02b	0.97b	0.61	1.22
pH value	3.97a	4.06a	4.03a	0.93	0.83
Mean PPFD (μmol m^−2^ s^−1^)	14.76a	27.33b	40.52c	**9.93**	**3.34**

Note

The different letters next to the values indicate significant differences at *p* < .01, based on Tukey's HSD test. The odds ratios are significantly different from 1 (*p* < .01) as indicated by the bold values.

### 3.5 Distribution pattern of advanced seedlings and saplings

**Figure 8:**
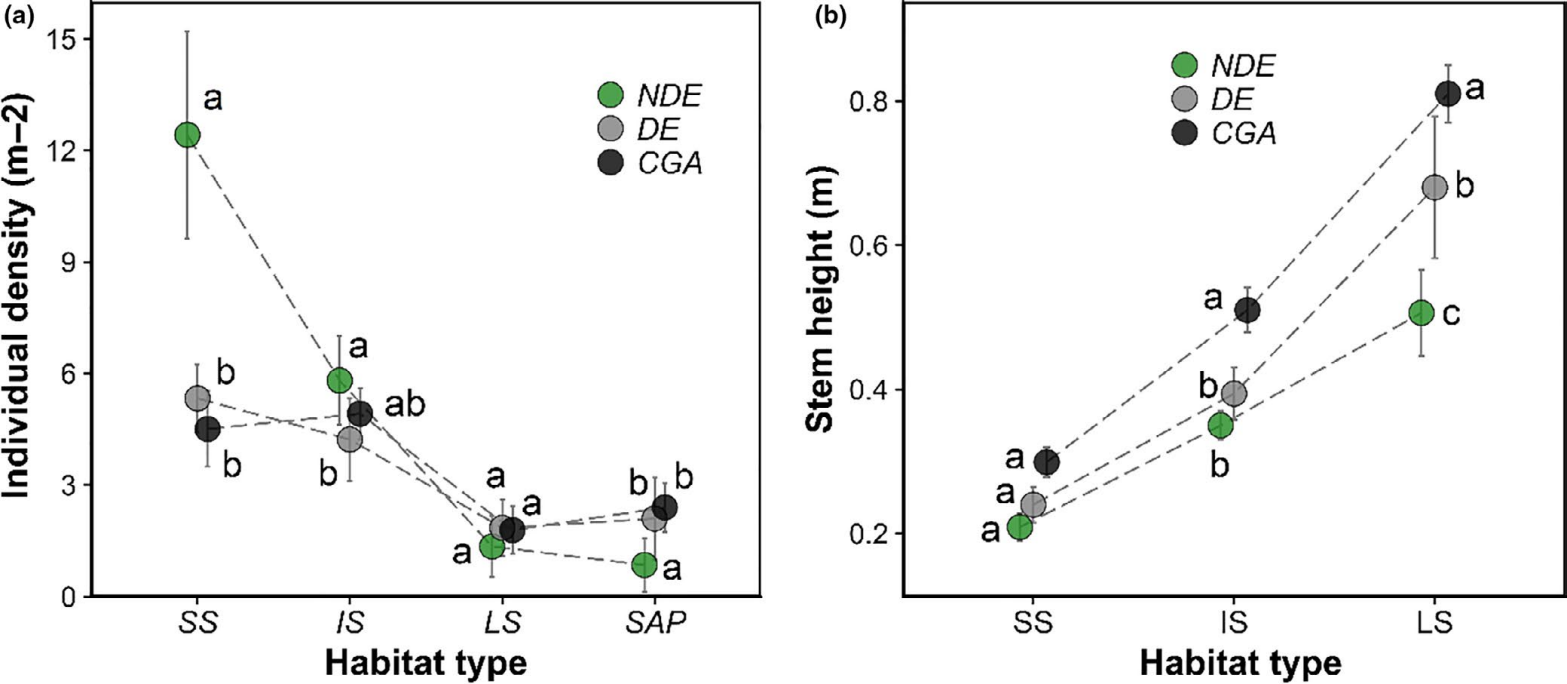
(a) Comparison of abundance and (b) height of recruits at different life stages in the three habitats. Error bars are for SE. The different letters above the dots indicate significant differences at *p* < .01, based on Tukey's HSD test. SS = small seedling; IS = intermediate seedling; LS = large seedling; SAP = sapling; NDE = under nondeclining canopies; DE = under declining canopies; and CGA = in canopy gaps

We recorded 1,247 advanced seedlings (small seedling, 650 individuals; intermediate seedling, 453 individuals; large seedling, 144 individuals) and 155 saplings (1.3–8 m in height) from the 30 subplots. Younger offspring, such as small seedlings, were most abundant in the NDE habitats. Older recruits, such as the saplings, were more abundant in the CGA habitats than in the NDE habitats (Figure 8a). Mean stem heights of intermediate seedlings and large seedlings in the DE and CGA habitats were also significantly larger than those in the NDE habitats (Figure 8b).

## 4 DISCUSSION

### 4.1 Pattern of decline of *Castanopsis fargesii*

In past decades, studies that addressed forest decline patterns were mainly concentrated on hardwood and *Pinus* species dominating the forests in North America and the Mediterranean basin (Galiano et al., 2013; Vilà‐Cabrera et al., 2013). In recent years, cases of *C. fargesii* decline have been seen in evergreen broad‐leaved forests in the Yangtze River Basin, such as on Tiantong Mountain in Eastern China (Song and Da, 2016). In this study, we began quantifying the decline pattern and canopy recovery trend of *C. fargesii* in a national nature reserve on Jinyun Mountain in Southwest China. We found that more than half of the *C. fargesii* adult trees in our study area were in decline. The small‐ and medium‐sized adult individuals showed a higher probability of decline (Figure 3), a pattern that resembles the reports on some instances of drought‐ and infestation‐induced forest declines (e.g., Vilà‐Cabrera et al., 2013). Nevertheless, the decline patterns of *C. fargesii* had some uncommon characteristics, at least in the study period from 2013 to 2018. In 2013, while half of the *C. fargesii* adult trees were in decline, the proportion of high level of decline and dead individuals was relatively low. In addition, a considerable number of nondeclining adult trees and saplings were maintaining the stability of the population structure. During the period of decline, nondecline *C. fargesii* individuals were still dominant in the community. More importantly, we revisited the study plots and re‐evaluated the decline level of all adult trees in 2018 and found that most of the nondecline individuals had not continued to decline, and some of the intermediate decline individuals had gradually restored their vitality (Figure 4). These phenomena suggest that the decline of *C. fargesii* adult trees can represent a short‐term forest disturbance.

### 4.2 Effects on seed production

At the seed rain stage, negative effects were seen from the decline in adult trees on seed production, similar to the situation of *Pinus sylvestris* and *Quercus crispula* decline (Vilà‐Cabrera et al., 2014; Nakajima & Ishida, 2014). Defoliation causes a reduction in the crown leaf area of a tree, which reduces its carbohydrate gain and limits seed production (Palacio et al., 2014). The seed abortion rate (proportion of immature seeds) of declining trees, however, was relatively lower than that of nondeclining trees, suggesting that they had a higher fruiting efficiency. In addition, the proportion of vertebrate‐attacked and insect‐attacked seeds of declining trees was lower than that of nondeclining trees, which suggests that their seeds had less survival pressure (e.g., vertebrate attack and insect attack) at the seed production stage. As a result, despite the reduction in absolute number of seeds, the intermediate decline individuals were still able to maintain a considerable seed production (over 60 seed/m^2^). However, the sharp reduction in seed production of high decline trees (2.1 seeds/m^2^) could have an adverse effect on the seedling regeneration in canopy gaps, at least during the seed rain stage.

Negative effects of adult tree decline on seed mass and nutritional traits were not found, suggesting that seed quality from the declining seeds did not decrease. Interestingly, the content of defensive materials (e.g., tannin and cellulose) of the seeds produced by declining trees was significantly lower than that of the nondeclining adult trees. Thus, not even the reduced defensive material content had a negative effect on seed survival at the seed production and seed dispersal stages (Figures 5 and 6). By combining these findings, except for severely declining individuals, the declining adult trees can still produce large numbers of mature seeds. More importantly, their key traits (i.e., potential germination capacity and competitiveness of seedlings) were not significantly reduced. In summary, no seed limitation was found in the declining adult trees. These results support Prediction 1.

### 4.3 Effects on new seedling recruitment

At the seed dispersal stage, rodent attack was the main seed‐killing agent, causing the death of most mature seeds in both nondeclining and declining habitats (Figure 6e). Rodent attack was a density‐dependent seed‐killing agent that caused seeds to face a higher risk of loss in vitality in the NDE habitats due to the higher seed density (Tomita et al., 2002; Zhu, Comita, Hubbell, & Ma, 2015). In contrast, seeds in the DE and CGA habitats had a higher survival rate due to their relatively low seed densities (Figure 7). Interestingly, a considerable number of seeds were transported into the CGA habitats by rodents, resulting in more viable seeds in the CGA habitats, compared to the input of mature seeds by seed rain (Figure 6b). Our recent research (unpublished seed dispersal experiment using 1,500 labeled seeds) also showed that about 5% of the total seeds under the canopies of the adult trees have been transported into forest gap habitats. These seed dispersals by rodents played an important role on seedling recruitment in the CGA habitats. At the seed germination stage, no significant difference was seen in the germination rate of viable seeds in the three types of habitats, indicating that the adult tree decline did not affect seed germination. Considering the whole seedling recruitment process, the seed to seedling transition rate in the DE and CGA habitats was significantly higher than that of the NDE habitats (9.47% and 109.24% vs. 7.94%; Figure 6d). In summary, the higher seed to seedling transition rate in the NE and CGA habitats, to a great extent, reduced the negative effects of seed production on new seedling recruitment. These results support Prediction 2.

### 4.4 Effects on seedling establishment

The survival and growth of new seedlings play an important role in seedling regeneration and community dynamics (Zhu et al., 2015). In this study, the survival rate and relative height growth of new seedlings in the DE and CGA habitats were enhanced relative to the NDE habitats (Figure 7). In addition, an abundance of advanced seedlings and saplings existed in the community. The younger recruits (e.g., small seedlings and medium seedlings) were concentrated in the NDE habitats, while the older recruits such as large seedlings and saplings were concentrated in the CGA and DE habitats (Figure 8a). Thus, the abundance of small seedlings does not mean a higher efficiency of seedling establishment in the NDE habitats since most of the seedlings could not survive due to the unfavorable understory environment (Yang et al., 2015; Zhu et al., 2015). In contrast, the few seedlings in the declining habitats, especially in the forest gaps, were more likely to occupy spaces generated by the death of adult trees due to the better understory environment. In total, even though the tree species is shade‐tolerant and late‐successional, the improved light conditions that follow a decline in *C. fargesii* can indeed promote the survival and growth of its seedlings. The higher seedling survival and growth rate in the CGA habitats further reduced the negative impact of drastic seed production reduction in adult trees decline on seedling regeneration. These results support Prediction 3.

## 5 CONCLUSION AND MANAGEMENT IMPLICATIONS

**Figure 9:**
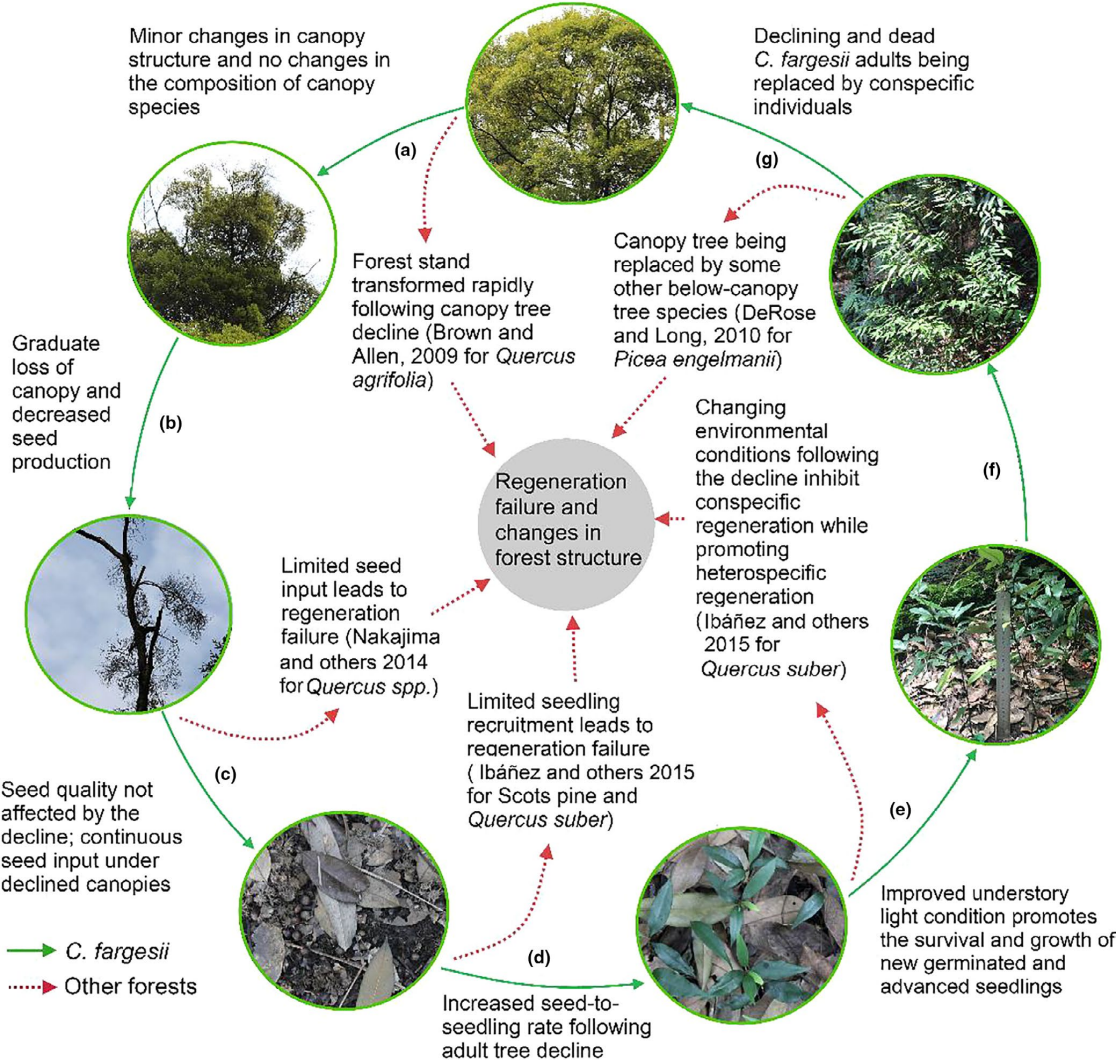
Effects of *C. fargesii* adult tree decline on conspecific regeneration at different stages of their life history, and the differences between *C. fargesii* decline and decline of some other dominant tree species, in terms of forest dynamics. (a) nondeclining adult tree; (b) intermediate level declining adult tree; (c) high level declining adult tree; (d) seeds; (e) new seedlings; (f) younger recruits; and (g) saplings

Successful regeneration postdecline was determined by multiple factors, such as decline intensity, seed production capacity, new seedling recruitment, seedling survival, and sapling growth (Brown & Allen‐Diaz, 2009; DeRose & Long, 2010; Ibáñez et al., 2015; Nakajima & Ishida, 2014). Regeneration failure can occur at any stage, causing the long‐term alteration of community species composition and forest structure (Figure 9). The prediction of regeneration trajectories of declining species is not an easy task. In this study, we found that *C. fargesii* decline has some special characteristics, compared to that of *Pinus* and hardwood species‐dominated forests, namely: (a) The decline of *C. fargesii* adult trees did not dramatically change the forest stand structure in the short term; (b) negative effects of adult tree decline were seen in seed production but no effects were seen on seed key traits; (c) although few seeds were seen in canopy gaps, the seed to seedling transition rate was relatively high due to the effective seed dispersal; (d) newly germinated seedlings in the canopy gaps and decline habitats had a higher survival rate and relative growth rate; and (e) a large number of well‐grown advanced seedlings were distributed in the forest gaps. These positive phenomena suggested that the short‐term defoliation and mortality of *C. fargesii* adult trees could be seen as a natural disturbance that promoted conspecific seedling regeneration. Hence, we predict that the canopy gaps occurring after adult tree mortality and decline will be occupied by conspecific individuals. Nevertheless, longer‐term monitoring is necessary to confirm that these trends are maintained through time or if other mechanisms might prevent *C. fargesii* regeneration.

The evergreen broad‐leaved forests were near‐climax and codominated by some late‐successional species such as *C. fargesii*, *Machilus pingii*, *Schima superba,* and *C. carlesii* var. *spinulosa* (Wang, Kent, & Fang, 2017). Moreover, the understory environmental condition of these forests was characterized by high humidity and low light intensity. The most stressful environmental condition that limited the establishment of seedlings occurred at the seedling stage. The regeneration and maintenance of these dominant tree species depended mostly on improvements in the local environmental conditions, such as fine‐scale disturbances and the creation of small gaps. In general, seed abundance and seed germination as limiting factors were not present for these species, even though some individuals were declining. There were abundant small seedlings under the crown of healthy adult trees. When the adult trees were in decline, these pre‐established seedlings will be released due to the improved environmental conditions. Thus, we regard the intermediate decline of the evergreen broad‐leaved forest as a forest disturbance that can promote the regeneration of its dominant species. We also believe that high‐intensity management measures would be unnecessary in cases of an emerging intermediate decline in this forest. Furtherly, the management of this mature forest could benefit from the creation of small gaps by cutting some early‐successional trees to improve the late‐successional species regeneration.

## CONFLICTS OF INTEREST

The authors declare no conflicts of interest.

## AUTHOR CONTRIBUTION

**Li Huang:** Conceptualization (Supporting); Writing—original draft (Lead). **Lihua Zhou:** Investigation (Supporting). **Jingmei Wang:** Investigation (Supporting). **Siwei Hu:** Investigation (Supporting). **Shenhua Qian:** Writing—original draft (Supporting). **Dunmei Lin:** Investigation (Supporting). **Liang Zhao:** Investigation (Supporting). **Yongchuan Yang:** Conceptualization (Lead).

## Data Availability

The data that support the findings of this study are available at the Dryad Digital Repository (https://doi.org/10.5061/dryad.dncjsxkx4).
